# Long-Term Favorable Outcome With Nivolumab in a Case of Advanced Non-Small Cell Lung Cancer: A Case Report

**DOI:** 10.7759/cureus.18526

**Published:** 2021-10-06

**Authors:** Vijay Ketan Reddy, Dhan B Shrestha, Suman Gaire, Wasey Ali Yadullahi Mir, Mohammed Kassem

**Affiliations:** 1 Department of Internal Medicine, Mount Sinai Hospital, Chicago, USA; 2 Department of Emergency Medicine, Palpa Hospital, Palpa, NPL; 3 Department of Hematology and Oncology, Mount Sinai Hospital, Chicago, USA

**Keywords:** non-small cell lung carcinoma, nivolumab, monoclonal antibodies, lung neoplasms, immunotherapy

## Abstract

Non-small cell lung cancer (NSCLC) constitutes around 85% of lung cancer cases. Advanced non-small cell lung cancer has a poor prognosis. Immunotherapy plays a pivotal role in managing advanced non-small cell lung cancer not positive for driver mutations. Nivolumab is a monoclonal antibody against programmed death-ligand 1 (PDL1). It is approved as a second-line treatment for patients with advanced non-small cell lung cancer who progress on or after chemotherapy. We present a case of a 71-year-old female with advanced non-small cell lung cancer without any driver mutations diagnosed four years ago. Her disease progressed while on conventional chemotherapy, and she was started on nivolumab three and a half years ago. Her lung nodules resolved, she did not show signs of progression, and her performance status improved while on nivolumab. This case report highlights the current role of nivolumab in the management of NSCLC. Patients whose condition worsens while on conventional chemotherapy can respond very well to modern targeted immunotherapy.

## Introduction

Lung cancer is the leading cause of cancer deaths worldwide and in the United States [1,2]. Non-small cell lung cancer (NSCLC) constitutes about 85% of lung cancer cases [3]. The prognosis of lung cancer is poor with a five-year survival of 10%-20% after the diagnosis. Despite the progress in treatment and survival in NSCLC, the two-year survival in distant metastatic NSCLC is still around 20% only [1]. There are targeted therapies for various driver mutations, such as epidermal growth factor receptor (EGFR), anaplastic lymphoma kinase (ALK), and receptor tyrosine kinase (ROS1), in NSCLC, which have improved the prognosis of patients with driver mutations [4,5]. For patients without driver mutations, immunotherapy has played a pivotal role in the management of NSCLC. Several immune checkpoint inhibitors targeting programmed death 1/programmed death-ligand 1 (PD1/PDL1) have been approved for NSCLC [4,5]. These immune checkpoint inhibitors have increased the survival of patients suffering from NSCLC [6].

Nivolumab is a monoclonal antibody against PDL1. It was approved for use in advanced NSCLC for patients who have progressed on or after platinum-based chemotherapy [5]. Compared with other PD1 inhibitors, nivolumab has demonstrated similar efficacy with a lesser incidence of severe adverse effects than pembrolizumab or atezolizumab [7].

We present a case of a 71-year-old female suffering from advanced NSCLC without any driver mutation who was managed with nivolumab.

## Case presentation

A 71-year-old female with no significant past medical history had presented four and a half years ago with a weight loss of about 50 pounds over six months prior to presentation. She complained of occasional shortness of breath but denied any cough. Physical examination revealed enlarged right axillary and supraclavicular lymph nodes.

Mammography was performed, revealing multiple enlarged right axillary lymph nodes, but no suspicious lesions were seen in either breast. An ultrasound-guided core biopsy of the right axillary lymph node was performed. The histopathology report showed poorly differentiated metastatic adenocarcinoma. The biopsy was cytokeratin 7 (CK7) and thyroid transcription factor-1 (TTF-1) positive and negative for CK20 and mammaglobin, respectively. Epidermal growth factor receptor (EGFR), ROS1, and anaplastic lymphoma kinase (ALK) were negative, and PD1 and PDL1 expressions were less than 1%.

**Figure 1:**
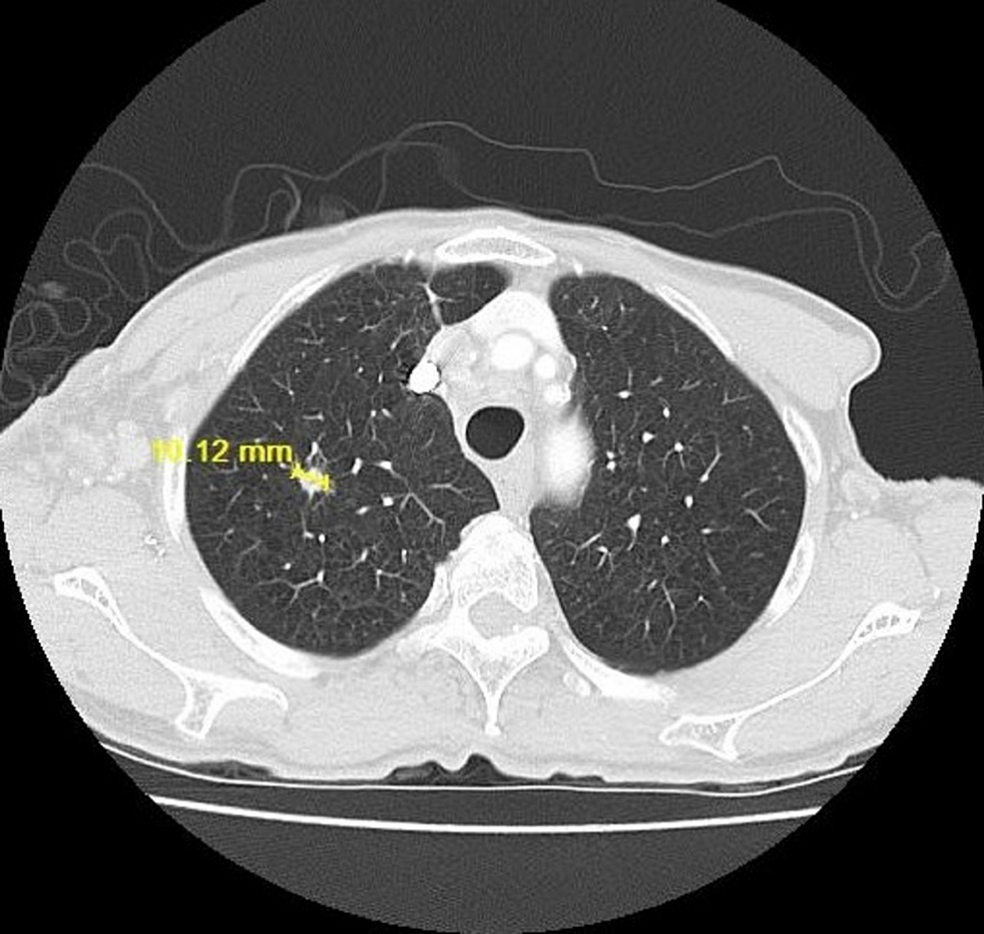
CT chest lung window showing right upper lobe nodule on 06/12/2017

**Figure 2:**
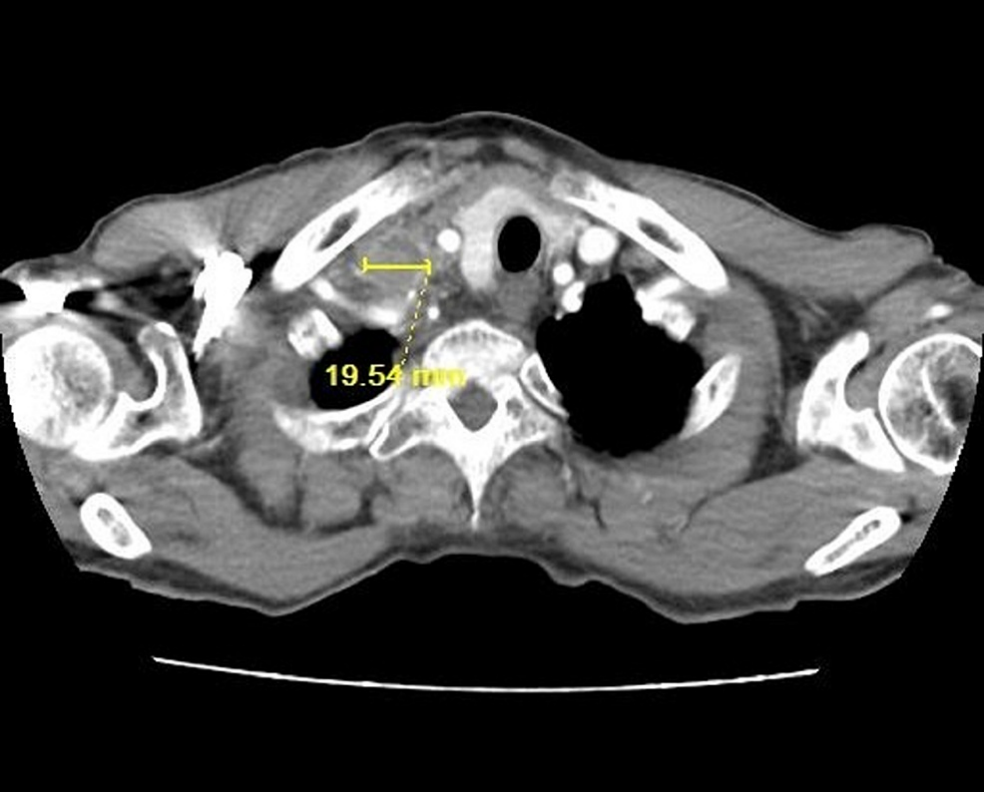
CT chest soft tissue window showing right subclavian lymph nodes, with the largest measuring 19.54 mm, on 06/12/2017

**Figure 3:**
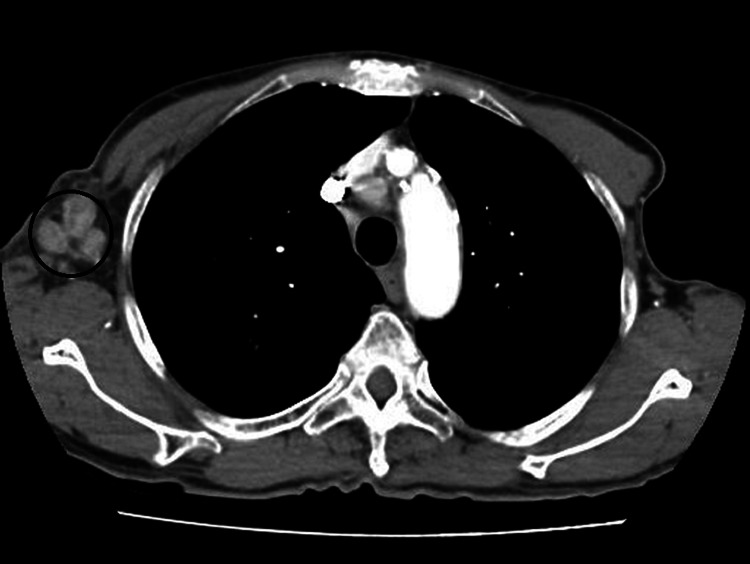
CT chest soft tissue window showing right axillary lymph nodes (marked with a black circle) on 06/12/2017

A computed tomogram (CT) of the chest with contrast enhancement was done. There were multiple irregular nodular opacities in bilateral lungs, with the largest measuring 10 mm, and extensive bilateral mediastinal lymphadenopathy and supraclavicular lymphadenopathy, with the largest measuring 1.8 cm (**Figures 1-3**). Magnetic resonance imaging (MRI) brain and bone scans were negative for any metastases. However, a positron emission tomography (PET) scan revealed hypermetabolic nodules in the lung and multiple suspicious nodes in the neck, chest, and abdomen, suggesting advanced stage IV lung cancer.

**Figure 4:**
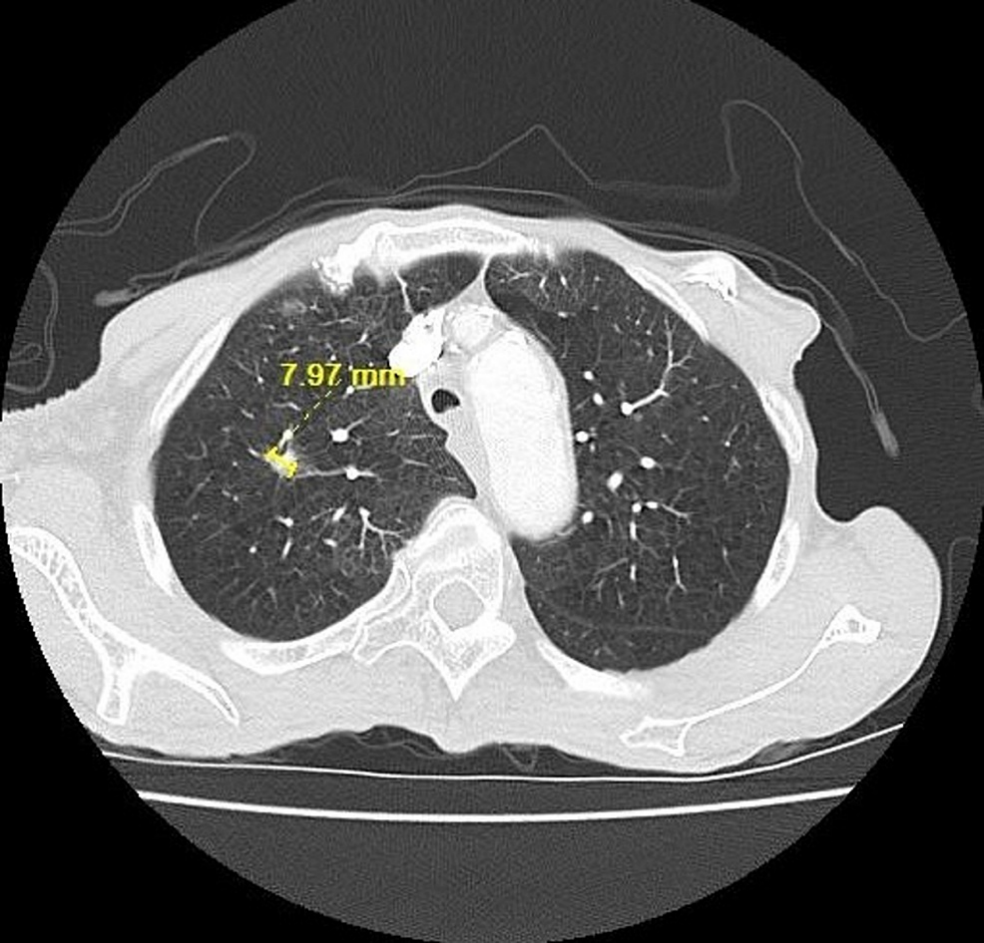
CT chest lung window showing right upper lobe lung nodule during chemotherapy on 11/27/2017

**Figure 5:**
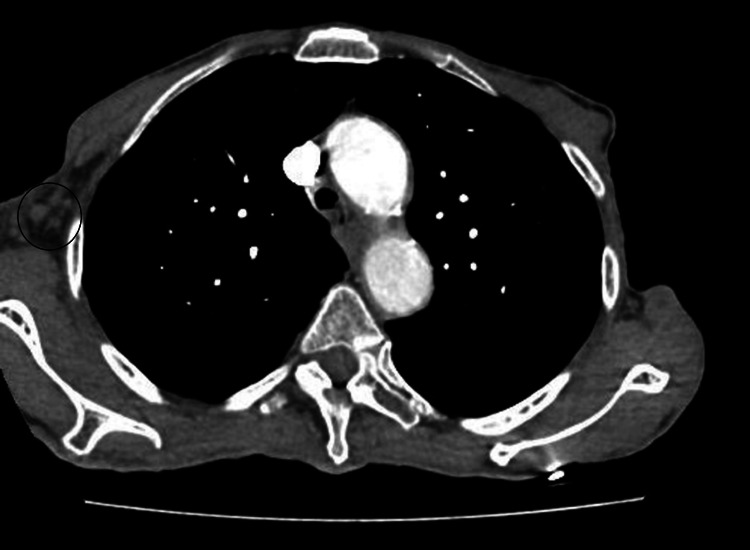
CT chest soft tissue window showing resolving enlarged right axillary lymph nodes (marked with a black circle) during chemotherapy on 11/27/2017

**Figure 6:**
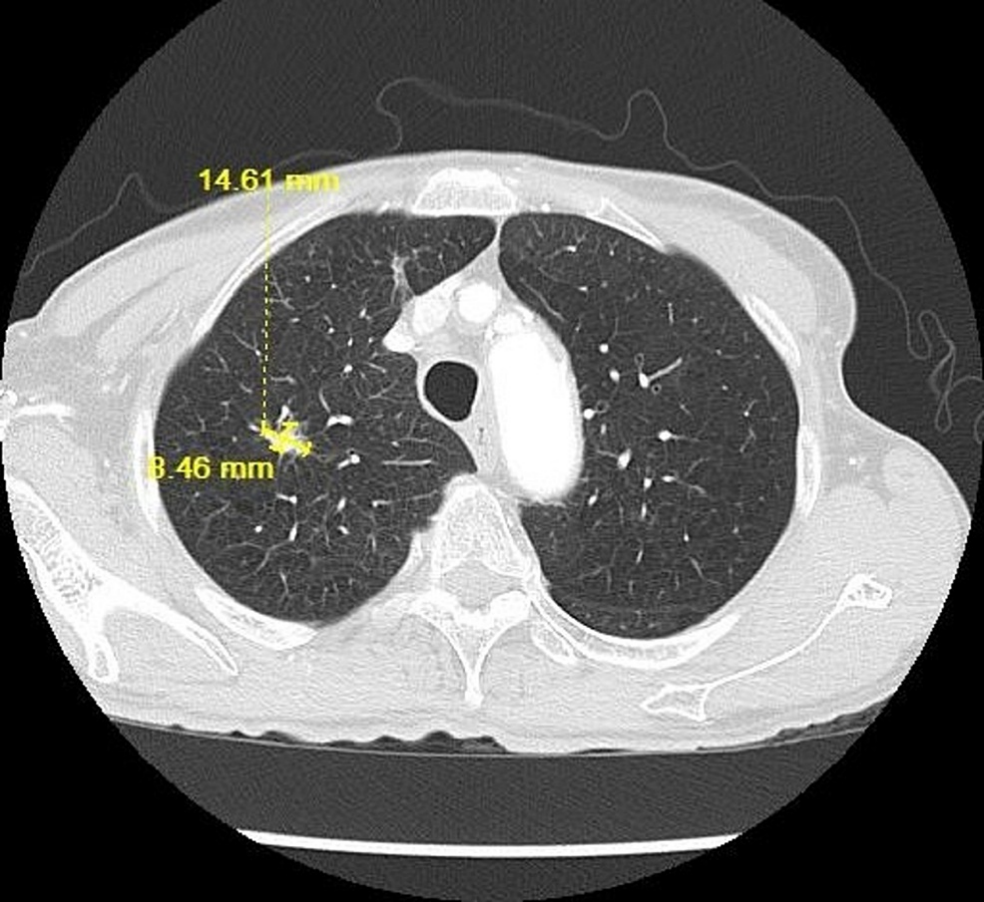
CT chest lung window showing the increased size of the lung nodule to 14.61 x 8.46 mm on 03/12/2018

**Figure 7:**
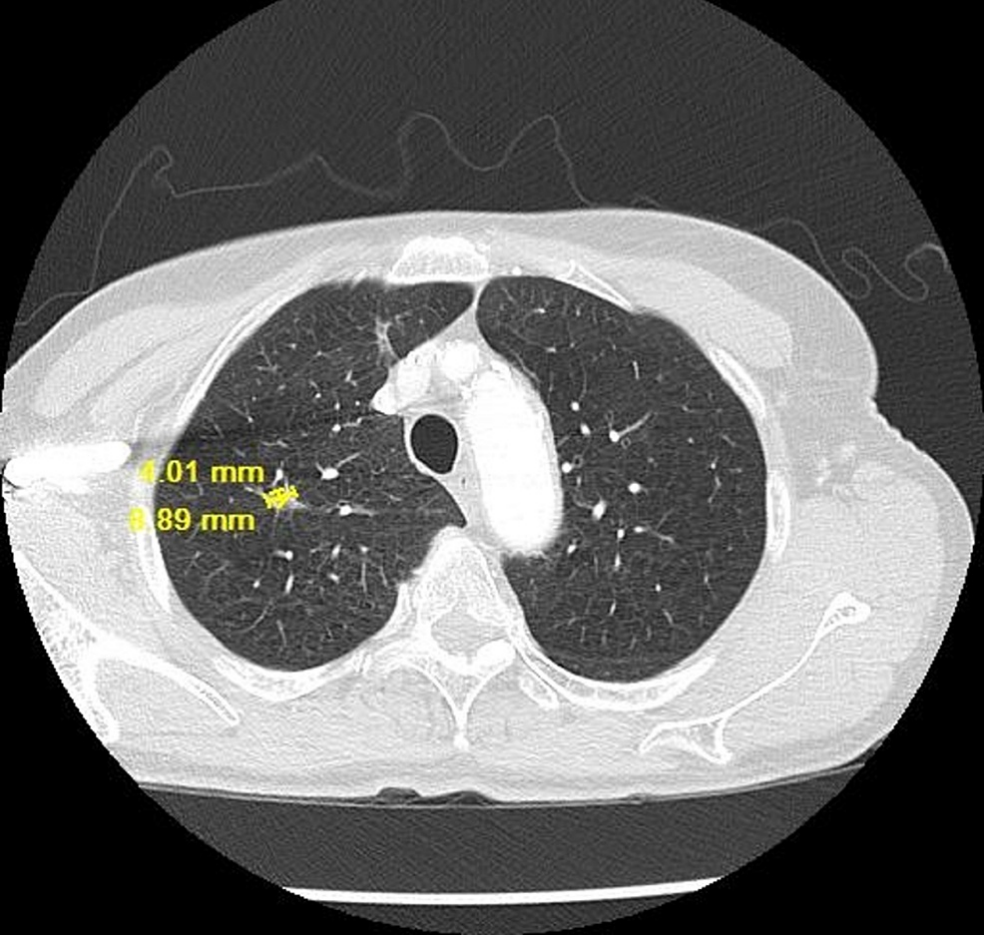
CT chest lung window showing resolving lung nodule on 10/26/2018

**Figure 8:**
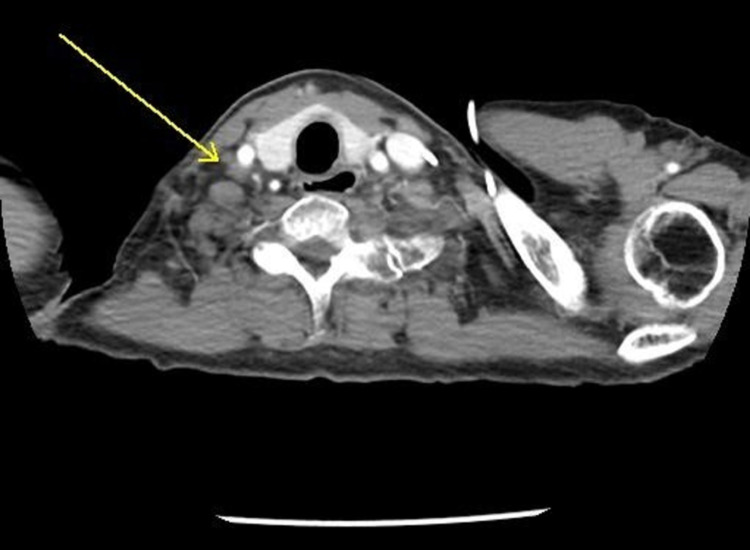
CT chest soft tissue window showing resolved right subclavian lymphadenopathy on 10/26/2018

**Figure 9:**
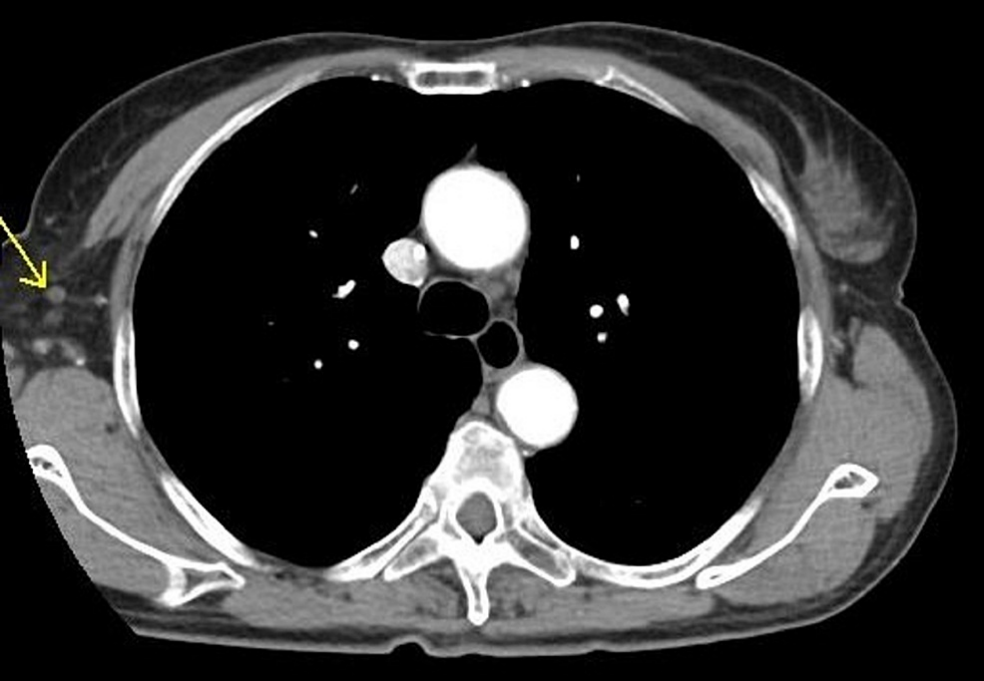
CT chest soft tissue window showing resolution of right axillary lymphadenopathy on 10/26/2018

**Figure 10:**
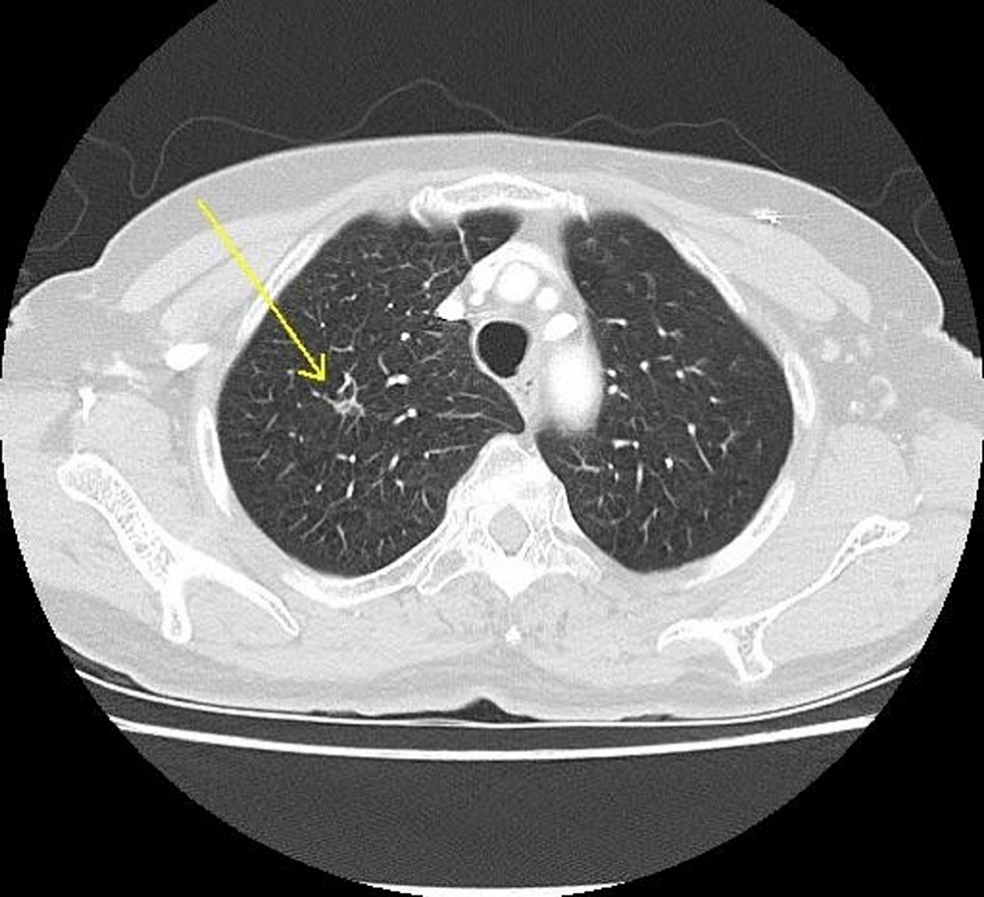
CT chest lung window showing stable lung field with a resolution of a prior nodule on 04/26/2021

She was initially treated with carboplatin, pemetrexed, and paclitaxel for a total of four cycles. She responded well to the treatment with a decrease in the size of pulmonary tumor nodules (from 11.12 to 7.97 mm) noted on the follow-up CT scan (**Figures 4-5**). She went on to complete eight cycles of maintenance pemetrexed, and on follow-up, a CT scan revealed the right upper lobe lung nodule to have increased in size from the previous 1 x 0.7 cm to 1.5 x 0.8 cm (**Figure 6**). At this time, she was started on nivolumab (3 mg/kg/dose). Six months following commencement of nivolumab, complete resolution of lung lesions was seen on CT chest (**Figures 7-9**). The lung lesions remain unremarkable since then (**Figure 10**). Her performance status was stable at ECOG-PS-0 over the four years of treatment, with weight gain from improvement in her appetite. She had resolution of all her respiratory symptoms and B symptoms.

After being on treatment for almost four years, the patient developed a pruritic, hyperpigmented rash on the upper and lower extremities and trunk. The rash was associated with dermographism and minimal erythema, suggesting grade II-III maculopapular rash, a well-known side effect often seen in patients being treated with checkpoint inhibitors. At this time, she received a treatment break from nivolumab while she was treated with steroids (1 mg/kg/day of prednisone) and hydroxyzine.

**Figure 11:**
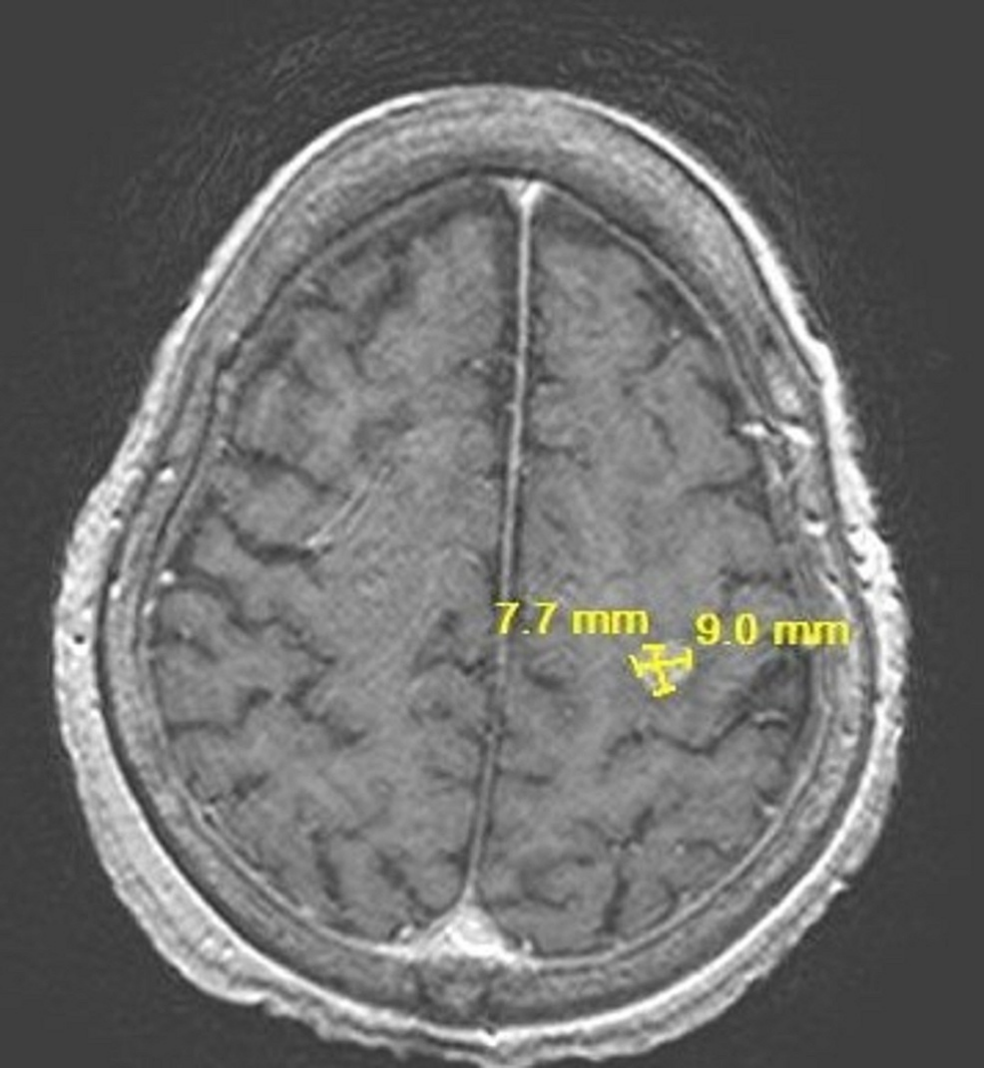
MRI brain showing incidental brain metastatic lesion on 07/08/2021

**Figure 12:**
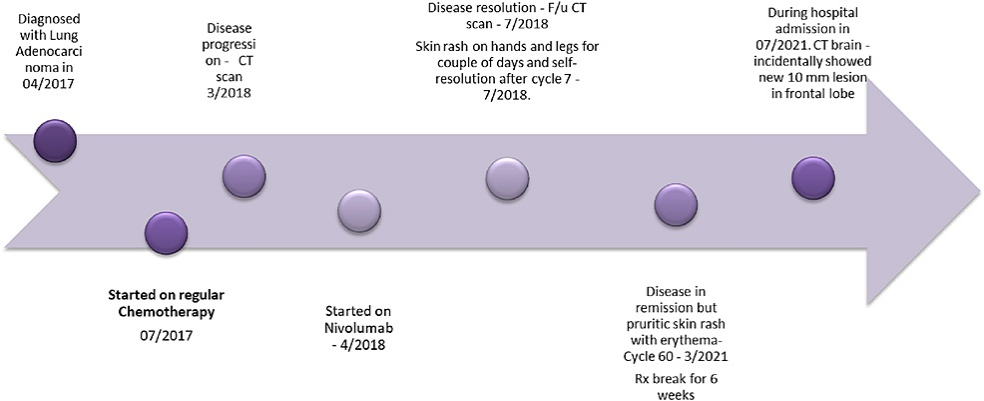
Timeline of the main events during the course of diagnosis and treatment

On the resolution of her rash, she was restarted on nivolumab. The patient presented to ER and was subsequently admitted for significant posterior epistaxis and melena along with a drop in her hemoglobin. A CT head scan was performed as part of the workup for severe posterior epistaxis, which showed a 10 mm enhancing lesion in the posterior left frontal lobe, suspicious for metastatic disease. MRI brain confirmed a 0.9 cm enhancing lesion at the posterior left frontal lobe (**Figure 11**). For the incidental metastatic lesion in her brain, she received stereotactic radiosurgery, given her solitary lesion. The timeline of the events is shown in **Figure 12**.

## Discussion

The treatment of advanced stage NSCLC is palliative in intent to prolong survival and maintain quality of life. With the advent of immunotherapy, immune checkpoint inhibitors have been incorporated into the first-line management of advanced NSCLC. Furthermore, immune checkpoint inhibitors such as nivolumab can be used in patients who progress on or during standard chemotherapy or those who did not receive checkpoint inhibitors as first-line therapy [8].

Our patient at diagnosis had stage IV adenocarcinoma of the lung. She received palliative chemotherapy, followed by pemetrexed maintenance therapy. However, she was started on nivolumab monotherapy due to subsequent disease progression.

Nivolumab is a monoclonal antibody against PDL1. Tumor cells in NSCLC express various neoantigens that can evade T cells and are sensitive to multiple immune checkpoint inhibitors [9]. PDL1 is expressed in lung cancer, ovarian cancer, colon cancer, and melanoma [10]. PDL1-PD1 interaction prevents the activated CD8 T cells from lysing their target cells and promotes CD8 T cell apoptosis [11]. Thus, antibodies that block PD1 or PDL1, such as nivolumab, potentiate the antitumor immune response.

The Checkmate 227 trial, which compared nivolumab/ipilimumab, nivolumab alone, and chemotherapy in patients suffering from NSCLC, reported higher overall survival, progression-free survival, and objective response rate in nivolumab/ipilimumab as compared with chemotherapy. The benefits were present regardless of PDL1 level [12]. The combination, therefore, can be used as first-line therapy. However, nivolumab alone is recommended only as second-line therapy. When used as second-line therapy for advanced NSCLC, nivolumab was found to have better efficacy in terms of progression-free survival (hazard ratios [HR]: 0.70, P = 0.03) and overall survival (HR: 0.70, P < 0.00001) as compared to docetaxel in a meta-analysis that pooled six studies [13]. In a real-world pooled analysis of 2585 patients, the median overall survival in previously treated patients with NSCLC on nivolumab was found to be 11.3 months (95% CI: 10.5-12.2) [14].

Our patient showed resolution of lung lesions on CT and no disease progression on nivolumab for three and a half years. However, the MRI brain scan showed a new solitary metastasis in the brain, which was treated with stereotactic radiosurgery, as nivolumab, similar to other monoclonal antibodies, are expected to have no blood-brain barrier penetration [15,16].

## Conclusions

Immunotherapy plays a vital role in the management of patients with NSCLC, especially those lacking driver mutations. Nivolumab is an effective second-line immunotherapeutic agent for advanced NSCLC regardless of PDL1 expression. For the management of NSCLC, nivolumab is used if the patient shows progression on or after chemotherapy. The case report shows the current role of nivolumab in the management of NSCLC. It also highlights the promise of immunotherapy in maintaining the quality of life and prognosis of driver mutation-negative NSCLC.
